# Context Attention Heterogeneous Network Embedding

**DOI:** 10.1155/2019/8106073

**Published:** 2019-08-21

**Authors:** Wei Zhuo, Qianyi Zhan, Yuan Liu, Zhenping Xie, Jing Lu

**Affiliations:** ^1^School of Digital Media, Jiangnan University, Wuxi 214122, China; ^2^Jiangsu Key Laboratory of Media Design and Software Technology, Wuxi 214122, China

## Abstract

Network embedding (NE), which maps nodes into a low-dimensional latent Euclidean space to represent effective features of each node in the network, has obtained considerable attention in recent years. Many popular NE methods, such as DeepWalk, Node2vec, and LINE, are capable of handling homogeneous networks. However, nodes are always fully accompanied by heterogeneous information (e.g., text descriptions, node properties, and hashtags) in the real-world network, which remains a great challenge to jointly project the topological structure and different types of information into the fixed-dimensional embedding space due to heterogeneity. Besides, in the unweighted network, how to quantify the strength of edges (tightness of connections between nodes) accurately is also a difficulty faced by existing methods. To bridge the gap, in this paper, we propose CAHNE (context attention heterogeneous network embedding), a novel network embedding method, to accurately determine the learning result. Specifically, we propose the concept of node importance to measure the strength of edges, which can better preserve the context relations of a node in unweighted networks. Moreover, text information is a widely ubiquitous feature in real-world networks, e.g., online social networks and citation networks. On account of the sophisticated interactions between the network structure and text features of nodes, CAHNE learns context embeddings for nodes by introducing the context node sequence, and the attention mechanism is also integrated into our model to better reflect the impact of context nodes on the current node. To corroborate the efficacy of CAHNE, we apply our method and various baseline methods on several real-world datasets. The experimental results show that CAHNE achieves higher quality compared to a number of state-of-the-art network embedding methods on the tasks of network reconstruction, link prediction, node classification, and visualization.

## 1. Introduction

Nowadays, information networks are ubiquitous in our daily life, for example, social and communication networks, citation networks, and co-occurrence networks. At most of the time, the scales of real-world networks are very large. Thus, analyzing large-scale networks has attracted considerable research attention in recent years. Network embedding (NE), also known as network representation learning, aims to generate informative numerical representations for nodes in the network to preserve network structures and further alleviates the inconveniences caused by sparsity. Network embedding methods are demonstrated to be effective in many network analysis tasks including link prediction [1], node classification [2], and clustering [3].

Many approaches have been proposed toward this goal, such as DeepWalk [4], LINE [5], Node2vec [6], and PPNE [7]. Particularly, network embedding aims to project the network into a low-dimensional space, where each node is represented using a corresponding embedding vector, and the relativity among nodes is preserved. The nodes with “high similarity” are mapped onto adjacent points (“high similarity” means nodes have similar properties and are more likely to have edges between them). The embedding vectors contain the semantic information transcribed from the network structure and can be applied in various network mining applications easily. However, most of the existing NE methods take the network structure as input to learn representations for nodes without considering any other information.

**Figure 1:**
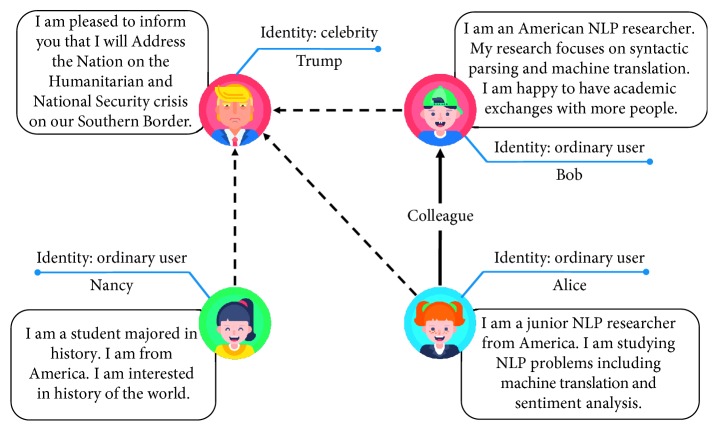
Example of relationships between users in Twitter and the content of their tweets. Dotted lines and solid lines represent weak connections and strong connections, respectively.

In reality, a network usually has rich heterogeneous information, such as text descriptions and other metadata. For instance, Wikipedia (https://www.wikipedia.org/) entries connect with each other and build an encyclopedia network. Simultaneously, each entry as a node has substantial text information such as keywords and introduction, which describe a node in detail and more comprehensively. Furthermore, in the real-world social network like Twitter (https://twitter.com) shown in Figure 1, users as nodes also have their own text descriptions, which may reflect the properties of each node. Hence, text information is typical and critical heterogeneous semantic information widely existing in real-world networks. However, most NE models treat all networks as homogeneous networks. In other words, most works learn representations only from network structures ignoring text information. Because of heterogeneity in networks, we put forward an idea to embed a network from both network structures and text information.

To this end, a direct way is to learn representations from text information of nodes and network structures independently, which can be called text-aware embedding. However, this way ignores the complicated interactions between network structures and text information, which leads to invalidity. CANE [8] is an efficient method to capture the correlation between the text feature of a node and its neighbors' in a network, which achieves the purpose we stated before. However, CANE only preserves the local relations in a network, while we need to take the global network structures into consideration rather than node pairs independently. For example, in Figure 1, Bob may have connections to other NLP researchers who are also his colleagues and Alice has not followed these researchers, so there may be potential relationships between these researchers and Alice in the text aspect because they have similar properties, but CANE cannot capture these relationships. Thus, how to satisfy the compatibility between network structures and text information in the network should be exploited to better represent nodes.

In addition to the problem stated above, typical NE methods are insensitive to the strength of the relationship between nodes in unweighted networks. As an intuitive example, we show some relationships from the real-world online networks in Figure 1. In Twitter, Trump is a celebrity who has plenty of followers, and each follower links to him by an edge. Alice and Bob are ordinary users, and they link with each other because they are colleagues. They also follow Trump just because they are Americans. In this case, the strength of the relationship between Alice and Bob should be stronger than that between Alice and Trump. As shown in Figure 1, we use dotted lines and solid lines to describe the strength of relationships (edges). Strong connection means high similarity between pairwise nodes, and weak connection means low similarity. In unweighted networks, classical NE methods generally treat the weight of the edge between nodes as a binary variable and ignore the rich semantics of edges we illustrated before. Therefore, the strength of connections is underlying structural information we need to take into consideration when learning network representations in real-world networks, which remains a great challenge.

From the aforementioned problems, the heterogeneity and structural complexity in real-world networks pose specific hurdles for network representation learning. Fortunately, in this paper, we propose a context attention heterogeneous network embedding (CAHNE) method with an emphasis on leveraging the rich and intrinsic information in heterogeneous networks. Specifically, CAHNE reconstructs the classical network represented as *G*=(*V*, *E*) to form the heterogeneous text network denoted as *G*=(*V*, *E*, *T*). We can extract a context node sequence for each node by breadth-first search (BFS) on the redesigned network, and the root node can be deemed the anchor node. Through a series of specific operations that we will give a detailed elaboration in the later section, combining the text information in a sequence, we can obtain a representation for the anchor of the context node sequence, which is the context embedding of the anchor node. Therefore, CAHNE integrates text information into the global structures of the network to learn the potential intertextual associations in the network. Moreover, the influence of context nodes on the anchor node can vary with different anchor nodes, and thus, we further the adopt attention mechanism to enhance the expressiveness of the influence from the context nodes on the specific anchor node. Besides, for unweighted networks, CAHNE is expected to preserve the underlying structural information on the strength of edges. Based on this idea, we give the definition of node importance that quantifies the strength of the relationship between nodes and integrate it into the network embedding method to learn a structure-based representation for each node. Finally, we concatenate the context embedding and the structure-based embedding of the node as the complete representation for the node. Empirically, we apply CAHNE to four network analysis tasks, i.e., network reconstruction, link prediction, node classification, and visualization, using seven real-world networks as datasets. Experimental results demonstrate that our method learns better nodes embeddings when compared to a variety of state-of-the-art baselines in the field of NE.

The main contributions of our method are summarized as follows:

- We propose a novel network embedding model, namely, CAHNE. The method is able to learn comprehensive representations for different types of real-world networks, which confirms the flexibility and robustness of our model.
- We provide a key insight regarding the strength of relationships in unweighted real-world networks. We thereby propose the definition of node importance for optimizing the objective, which more closely shows the actual situations of the network.
- We integrate heterogeneous information into network representation and mitigate the incompatibility between network structures and text information by extracting context node sequences accompanied by the attention mechanism to learn context embeddings.

The source code is available at https://github.com/zhuo931077127/CAHNE.

## 2. Related Works

Network representation learning (NRL) has been well researched for many years, for example, in earlier works such as Isomap [9], multidimensional scaling (MDS) [10], and Laplacian eigenmap (LE) [11]. These approaches represent the network as an affinity graph by using the feature vectors of the network nodes. For a given large-scale information network, e.g., social network and citation network, these methods are less efficient and inflexible to generate node representations.

In recent years, inspired by the development of the machine learning and word embedding method Word2vec [12], many NRL methods have been proposed for large-scale information network representation. For example, DeepWalk [4] proposes to perform random walks on the graph to obtain sequences of nodes. It introduces the Skip-Gram model to achieve vertex representations. Based on DeepWalk, Node2vec [6] defines a flexible notion of a node's network neighborhood and designs a biased random walk procedure to explore the network structure more efficiently. Some other methods focus on finding multivariate structure features in the network. For example, LINE [5] embeds the network into a low-dimensional latent space to approximate the first-order proximity and second-order proximity of the network. Nevertheless, most of these network embedding models only focus on homogeneous networks, without taking heterogeneous information into consideration.

Different from homogeneous networks, heterogeneous networks consist of complex node and edge attributes. Several attempts have been done on heterogeneous information network (HIN) embedding and achieved promising performance in various tasks. Hin2Vec [13] learns the embeddings of a HIN by conducting multiple prediction training tasks jointly. CANE [8] learns network embeddings from network structures and text descriptions with mutual relations of pairwise nodes. ANRL [14] proposes a neighbor enhancement autoencoder to incorporate both the network structure and node attribute information in a principled way. Paper2vec [15] aims to learn the paper node embeddings from the paper citation network.

In summary, existing methods in homogeneous network embedding use either affinity matrix models or deep models to preserve network structural features in a low-dimensional space. And existing HIN embedding methods focus on different types of heterogeneous information. They have been proven useful on network analysis, but they cannot maintain the sophisticated interaction between network structures and heterogeneous information (in this paper, we consider text information). Additionally, to the best of our knowledge, all existing NE models ignore the important relationship information between nodes in unweighted real-world networks we proposed before. In contrast, our proposed model CAHNE can learn more comprehensive information than existing methods.

## 3. Preliminaries

In this section, we introduce basic definitions and formalize the problem of context attention heterogeneous network embedding.

### 3.1. Context Node Sequence (CNS)

**Figure 2:**
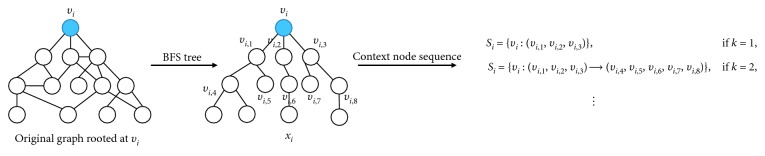
Example of the generating strategy for a context node sequence. The blue node is an anchor node *v*_*c*_.

Forming a context node sequence for the anchor node in the network can be viewed as a sampling process of detecting nodes that most likely have impact on the anchor node.  Figure 2 shows the process of obtaining a context node sequence. Concretely, we first perform breadth-first search (BFS) on the original graph *G* starting from a node *v*_*i*_ ∈ *V*, and we regard *v*_*i*_ as an anchor node, which provides us with a BFS tree *x*_*i*_ rooted at *v*_*i*_. *x*_*i*_ can be considered the unique relational tree of *v*_*i*_. Context nodes are not only the neighborhood of the anchor node but also deeper layer nodes. Hence, we control the number of layers by setting the parameter *k* to sample context nodes. Furthermore, the value of *k* is uncertain and determined by the type of the given network. At last, for a given node *v*_*i*_, we can obtain its context node sequence *S*_*i*_={*v*_*i*_ : (*v*_*i*,1_, *v*_*i*,2_, ⋯, *v*_*i*,*m*_)⟶(*v*_*i*,*m*+1_, ⋯, *v*_*i*,*m*+*n*_)⟶⋯}, where *m* and *n* are the number of context nodes in the first layer and second layer, respectively, and so on. *v*_*i*_ can also be treated as *v*_*i*,0_. It is worth noting that each node can only appear once or 0 times in a context node sequence and building BFS trees for all nodes is not computationally expensive because of the sparsity of real-world networks.

### 3.2. Problem Formulation

Now, we formally define the problem of CAHNE. Compared to conventional homogeneous network embedding such as DeepWalk and Node2vec, which only focus on a single network structure, our goal is to learn a representation for each node in a network graph with convergence of more heterogeneous associated information. Text information is widely available in real-world networks, e.g., social networks and citation networks, so we integrate it into the traditional graph definition (*G*=(*V*, *E*)) [16]. We first define a heterogeneous text network as follows.

**Definition 1 (heterogeneous text network (HTN)).**

The HTN is denoted as *G*=(*V*, *E*, *T*), where *V*={*v*_1_,…, *v*_*V*_} represents the set of nodes, *E*={*e*_*ij*_}_*i*,*j*=1_^|*V*|^ represents the set of edges, and *e*_*ij*_ is the relationship between two nodes (*v*_*i*_, *v*_*j*_) linked with each other, with an associated weight *w*_*ij*_ (in this paper, we only consider unweighted networks). *T*={*t*_1_, *t*_2_,…, *t*_|*V*|_} denotes the text information of nodes. For the text information of a specific node *v*_*c*_ ∈ *V*, we can represent it as a word sequence *t*_*c*_={*w*_1_, *w*_2_,…, *w*_*n*_*c*__}, where *n*_*c*_=|*t*_*c*_| denotes the number of words in *t*_*c*_.

Noticing the difference between the definition of the heterogeneous text network *G*=(*V*, *E*, *T*) and conventional network *G*=(*V*, *E*), the heterogeneous text network contains richer information. Empirically, weight often indicates the strength of the edge between two nodes. In practice, for unweighted real-world network datasets, weights are only formed as binary variables. For example, if *v*_*i*_ has a neighbor *v*_*j*_, the weight of the edge between them is 1; otherwise, it is 0. However, we expect to measure the strength of the relations more in line with the actual situations of real-world online networks. Thus, we propose the definition of node importance as follows.

**Definition 2 (node importance).**

Node importance is denoted as NI, which is a quantitative representation for each node in the network. It measures the strength of the edge between a given node and its neighbors. For an anchor node *v*_*i*_ ∈ *V*, NI(*v*_*i*_) is the value of node importance for *v*_*i*_.

In real-world networks such as citation networks and social networks, each node has its own context node sequence. We can integrate all nodes' CNSs and get a global sequence for *G*, *S*_*G*_=(*S*_1_, *S*_2_,…, *S*_|*V*|_). The more the CNSs a node consists of, in other words, the more the times a node appears in *S*_*G*_, the less the importance for this node to its neighbors. For instance, in Twitter, a celebrity has thousands of followers, which means this celebrity consists of abundant CNSs. However, for ordinary users, the importance of the relationship with a celebrity is less than that with their real friends who have relationships with them.

**Definition 3 (network embedding).**

Given a heterogeneous text network denoted as *G*=(*V*, *E*, *T*), network embedding aims to map the network data into a low-dimensional latent space, where each node *v* ∈ *V* can learn a low-dimensional embedding *v* ∈ *ℝ* ^*d*^ according to its graph structure and other information. Note that *d* ≪ |*V*| is the dimension of the latent embedding space.

Embedding a network into a low-dimensional space is helpful for many analysis tasks. In this process, the structures and properties of the network are preserved and encoded. In a heterogeneous text network, structure-based network embedding is not enough and the heterogeneous information is usually highly correlated with the network structure. Thus, we further propose the definition of context embedding.

**Definition 4 (context embedding).**

Aiming to learn a vector representation for the text information of each node in an HTN, context embedding learns a mapping function *f* : *t*_*i*_⟶*t*_*i*_ ∈ *ℝ*^*d*_*c*_^ for a node *v*_*i*_ ∈ *V*, where *d*_*c*_ is the dimension of context embedding.

It is worth mentioning that more than integrating text features of the anchor node, it also takes the context node sequence into consideration. For instance, the context embedding of the anchor node *v*_*c*_ is determined by its CNS *S*_*c*_ and its own text description *t*_*c*_. In this paper, our method CAHNE introduces the attention mechanism to weight the context nodes for each anchor node so that we can mitigate the incompatibility between network topologies and text features to obtain more comprehensive and accurate representations for the network.

## 4. CAHNE: The Proposed Method

In this section, we will give a detailed introduction to our method CAHNE.

### 4.1. Overall Framework

For CAHNE, we need to take full use of network structures and associated text information. We propose two types of embedding for a node *v* ∈ *V*, i.e., structure-based embedding *v*^*s*^ and context embedding *v*^*c*^. Structure-based embedding can capture the network structural information, which incorporates node importance, while context embedding can capture the textual meanings of anchor nodes accompanied by their context node sequences' text information. We concatenate two types of embeddings and obtain the overall node embedding for a node as follows:

(1) $$\begin{array}{c} v=v^{s}\;⊕\;v^{c}, \end{array}$$

where ⊕ indicates the concatenation operation. In the following sections, we will give a detailed introduction to the two types of embeddings, respectively.

### 4.2. Structure-Based Embedding

Without loss of universality, we assume the heterogeneous text network is directed. For the undirected network, we consider two directed edges with opposite directions and equal weights. And then, CAHNE fuses node importance as the weight for each node in the network.

#### 4.2.1. Node Importance

As noted in Definition 2, in a realistic network, the more the times a node appears in sequence *S*_*G*_, the less the importance to its neighbors. The quantitative representation of the importance of a node is the product of two statistics, node frequency (NF) and inverse CNS frequency (ICF). The node frequency refers to the frequency of a given node that appears in a context node sequence, which is a binary variable. In order to get the node frequency of *v*_*i*_, first we denote *f*_*ij*_ as whether *v*_*i*_ constitutes *S*_*j*_, where *v*_*j*_ ∈ *V*:

(2) $$\begin{array}{c} \begin{array}{c} f_{ij}=\left\{\begin{array}{cc} 0, & v_{i}\notin S_{j},\\ 1, & v_{i}\in S_{j}. \end{array}\right. \end{array} \end{array}$$

We denote *f*_*S*_*j*__ as the total number of nodes in the sequence *S*_*j*_. And then, we define NF(*i*, *j*) as the node frequency of *v*_*i*_ in *S*_*j*_, which can be formulated as NF(*i*, *j*)=*f*_*ij*_/*f*_*S*_*j*__.

ICF can be considered a measure of the universal importance of a node because it captures the distribution of importance in real-world networks. For a given node *v*_*i*_, we can denote ICF(*i*) as the inverse CNS frequency as follows:

(3) $$\begin{array}{c} \text{ICF}\left(i\right)=\log\frac{\left|V\right|}{\left\{j:v_{i}\in S_{k}\right\}}, \end{array}$$

where *k* ∈ {1,2,…, |*V*|}. After incorporating the mentioned node frequency and inverse CNS frequency, the node importance (NI) of a given node *v*_*i*_ can be measured as

(4) $$\begin{array}{c} \text{NI}\left(i\right)=\log\frac{\sum_{j}^{\left|V\right|}\left(\text{NF}\left(i,j\right)\cdot \text{ICF}\left(i\right)\right)}{\left|V\right|}. \end{array}$$

Note that NI is a context-based measure for each node in the network, and it extends TF-IDF thinking to network node analysis. Compared with the degree-based PageRank [17], NI incorporates richer contextual semantic structures rather than pairwise nodes, which enables our model to measure the importance of a node in the high-order neighborhood [18].

For a node *v*_*i*_ in an unweighted network, NI(*i*) can be served as the weights of edges starting from *v*_*i*_. We can also consider NI as the ranking of node popularity in the network. The smaller the value, the higher the prevalence of a node. After obtaining the quantitative representations of NI in a given network, we can simply obtain the empirical distribution of the network, which can be defined as follows:

(5) $$\begin{array}{c} \hat{p}\left(i\right)=\frac{\text{NI}\left(i\right)}{\sum_{v_{j}\in V}\text{NI}\left(j\right)}. \end{array}$$

#### 4.2.2. Structure-Based Objective

Formally, we model the conditional probability of *v*_*j*_ generated by *v*_*i*_ as

(6) $$\begin{array}{c} p\left(v_{j}\;|\;v_{i}\right)=\frac{\exp\left(v_{j}^{s}\cdot v_{i}^{s}\right)}{\sum_{v_{z}\in V}\exp\left(v_{z}^{s}\cdot v_{i}^{s}\right)}. \end{array}$$

This equation can be interpreted as the probability of detecting the edge from *v*_*i*_ to *v*_*j*_, which denotes the reconstructed distribution.

With the empirical distribution of the coincident probability between nodes and the reconstructed distribution, to preserve the node importance and network structures, a straightforward way is to minimize the following objective function:

(7) $$\begin{array}{c} O=\text{distance}\left(\hat{p}\left(\cdot \right),p\left(\cdot \right)\right), \end{array}$$

where distance(·, ·) is the distance between the two distributions. We choose KL divergence of two probability distributions to measure the difference between distributions. Thus, replacing distance(·, ·) with KL divergence, we can obtain the following objective:

(8) $$\begin{array}{c} {ℒ}_{s}\left(e_{ij}\right)=\text{KL}\left(p\;\|\;\hat{p}\right)=\sum_{\left(v_{i},v_{j}\right)\in E}p\left(v_{j}v_{i}\right)\text{log}\left(\frac{p\left(v_{j}\;|\;v_{i}\right)}{\hat{p}\left(i\right)}\right)\\ \propto -\sum_{\left(v_{i},v_{j}\right)\in E}\text{NI}\left(i\right)\text{log }p\left(v_{j}\;|\;v_{i}\right). \end{array}$$

With this formulation, we can minimize the objective equation (8) to obtain vectors {*v*_*i*_}_*i*=1..|*V*|_ ∈ *ℝ*^*d*_s_^ that represent nodes in the *d*_s_-dimensional latent space based on the network structure. We summarize the structure-based embedding method in Algorithm 1.

### 4.3. Context Embedding

CAHNE is expected to integrate typical heterogeneous information like text features in the network. A straightforward way is to learn representations from text information of nodes and network structures independently. However, it ignores the complex interactions and associations between topological structures and heterogeneous information. To bridge this gap, we introduce context embedding to fuse information of context nodes for an anchor in the network so that we can overcome the incompatibility problem.

As shown in Figure 2, we sample context nodes for the anchor node *v*_*i*_ and obtain a context node sequence *S*_*i*_ when setting *k* as 2. In a CNS, text features of different context nodes have various impacts on the anchor node. Thus, we expect to give a weight to each context node in a CNS, and the weights can reflect the impact trend of context nodes. To this end, we introduce exponentially weighted moving average [19].

#### 4.3.1. Exponentially Weighted Moving Average (EWMA)

Moving average (MA) is a calculation to analyze sequential data which reflect the changing trend in the sequence. Based on MA, exponentially weighted moving average (EWMA) applies weighting factors which decrease exponentially. The older data are attached with lower weights, but weights never reach zero. The EWMA for a sequence *Y* can be formulated recursively:

(9) $$\begin{array}{c} \text{EWMA}\left(t\right)=\gamma \text{EWMA}\left(t-1\right)+\left(1-\gamma \right)y\left(t\right)\\ =\left(1-\gamma \right)y\left(t\right)+\gamma^{2}\text{EWMA}\left(t-2\right)+\gamma \left(1-\gamma \right)y\left(t-1\right)\\ \vdots \\ =\overset{t}{\underset{i=1}{\sum }}\gamma^{t-i}\left(1-\gamma \right)y\left(i\right), \end{array}$$

where *γ* is a parameter that represents the degree of weight decrease and 0 ≤ *γ* < 1. *y*(*t*) is the current data, and EWMA(*t*) represents the EWMA value of the current data. In the tree *x*_*i*_, the deep layer nodes need to be given small weights because they are farther away from the anchor node. As a result, we can attach weight for each context node in *S*_*i*_. However, the nodes in the same layer need to be sorted first. For consistency, we sort the same layer nodes according to their NI values. And then, a normalized context node sequence can be generated for the anchor node *v*_*i*_ as *S*_*i*_={*v*_*i*_ : (*v*_*i*,1_, *v*_*i*_2__, ⋯, *v*_*i*,*e*_)}, where (*v*_*i*,1_, ⋯, *v*_*i*,*e*_) are sampled context nodes of *v*_*i*_. Afterwards, we apply EWMA on the context nodes from *v*_*i*,1_ as follows:

(10) $$\begin{array}{c} \text{EWMA}\left(v_{i,t}\right)=\gamma \text{EWMA}\left(v_{i,t+1}\right)+\left(1-\gamma \right)v_{i,t}. \end{array}$$

As the similarity we introduced EWMA, we treat *γ*^*t*−1^(1 − *γ*) as the weight of the context node *v*_*i*,*t*_, which is denoted as *𝒲*_*i*,*t*_.

#### 4.3.2. Text Information Representation

With the development of deep learning, there are many neural network models to learn text representations, e.g., convolutional neural network (CNN) [8, 20, 21], recurrent neural network (RNN) [22], long short-term memory (LSTM) [23], and gated recurrent units (GRUs) [24]. In this paper, we investigate different Word2vec models and find the CNN has the best performance on our tasks, which can capture comprehensive semantics in the heterogeneous text network.

**Figure 3:**
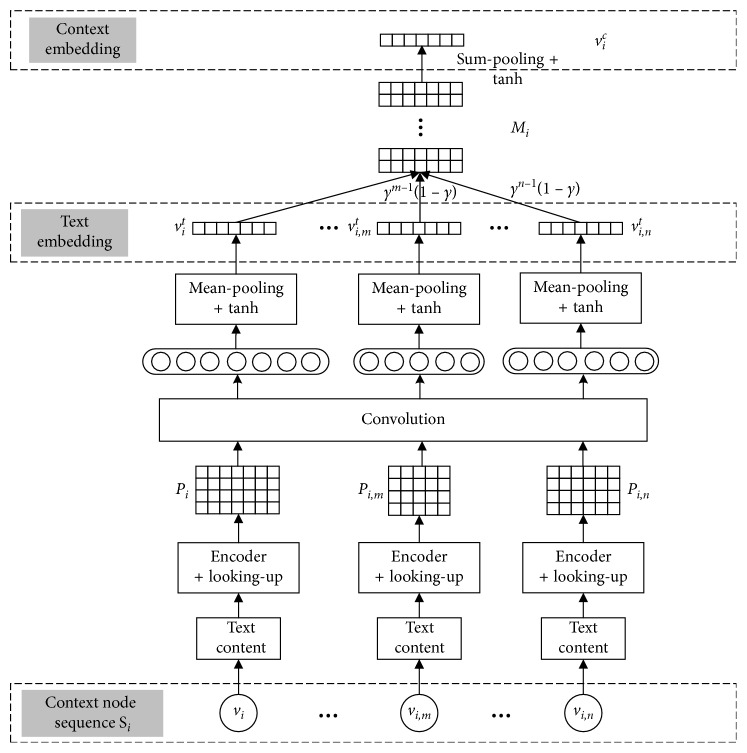
An illustration of context embedding for the anchor node *v*_*i*_.

In Figure 3, we show the framework of a generating process of context embedding. Given a normalized context node sequence *S*_*i*_ rooted at *v*_*i*_, we take the word sequence of each node in *S*_*i*_ as the input, and the CNN obtains text embedding through three layers, i.e., encoder and looking-up, convolution, and mean-pooling. And then, we adopt weighted summations for the representation vectors of the anchor node and its context nodes to obtain context embedding *v*_*i*_^*c*^ for *v*_*i*_.

*(1) Encoder and Looking-Up*. First, we map all words in the heterogeneous text network to a sequence of word IDs. Hence, we can obtain an ID sequence for *t* ∈ *T*. And then, the looking-up layer transforms each word *w* ∈ *t* into a vector *w* ∈ *ℝ*^*d*_*w*_^, where *d*_*w*_ is the dimension of word embeddings. Finally, we can obtain an embedding sequence *W*_*i*_=(*w*_1_,…, *w*_*n*_*i*__) for *v*_*i*_. As is shown in Figure 3, after the encoder and looking-up layer, we can get a matrix sequence *P*(*i*)=(*P*_*i*_,…*P*_*i*,*m*_,…, *P*_*i*,*n*_), and *P*_*i*_ is equivalent to *P*_*i*,0_.

*(2) Convolution*. After the encoder and looking-up layer, we use the convolution layer to extract the features of the input matrix sequence *P*(*i*). We perform convolution operation by a kernel *K* ∈ *ℝ*^*d*_t_×(1 × *d*_w_)^ to slide row by row in *P*_*i*,*x*_ (*x* ∈ {0, ⋯, *n*}) as follows:

(11) $$\begin{array}{c} y_{i,x}=K\cdot P_{i,x}+b, \end{array}$$

where *y*_*i*,*x*_=[*y*_1_^*x*^,…, *y*_*n*_*x*__^*x*^] denotes the feature vector of *P*_*i*,*x*_, in which *n*_*x*_ is the number of words in *t*_*i*,*x*_ (the text of *v*_*i*,*x*_), and *b* is the bias vector.

*(3) Mean-Pooling*. We test different pooling regulations. To get full-scale features of the text information for a node, we perform mean-pooling to get the text embedding *v*^*t*^. Then, we choose tanh as the nonlinear activation function over *y*_*i*,*x*_, which is

(12) $$\begin{array}{c} a_{j}=\tanh\left(\text{mean}\left(y_{1}^{x},\ldots ,y_{n_{x}}^{x}\right)\right), \end{array}$$

where *j* ∈ {1,2,…, *d*_*t*_}, in which *d*_*t*_ is the dimension of text embedding. At last, we can get the embedding of the text information for *v*_*i*,*x*_ as *v*_*i*,*x*_^*t*^=[*a*_1_,…, *a*_*d*_*t*__].

So far, we have obtained text embedding by the CNN for each node in a context node sequence. Following this, we do weight summations on the context node embeddings (*v*_*i*,1_^*t*^, ⋯, *v*_*i*,*n*_^*t*^), and this operation is sum-pooling in Figure 3. The strategy of generating context embedding for *v*_*i*_ is as follows:

(13) $$\begin{array}{c} v_{i}^{c}=\tanh\left(v_{i}^{t}+\overset{n}{\underset{j=1}{\sum }}W_{i,j}v_{i,j}^{t}\right). \end{array}$$

Through the method stated, we establish correlations between the anchor node and its context nodes in terms of representation vectors and maintain text relevance. Eventually, we can get context embedding for a given node *v*_*i*_, and the whole representation of *v*_*i*_ is bespoken as *v*_*i*_=*v*_*i*_^*s*^  ⊕  *v*_*i*_^*c*^.

The text embedding part of the context embedding framework shown in Figure 3 looks like the convolution method of CANE. The difference is that the input of our model is the CNS of a node, while the input of CANE is a pair of nodes. In addition, we sort the nodes in the CNS according to NI and weight each node in CNS with EWMA values, as shown in equation (13).

#### 4.3.3. Context Embedding Objective

Context embedding objective aims to measure the log-likelihood of a given directed edge (*v*_*i*_, *v*_*j*_) ∈ *E* as

(14) $$\begin{array}{c} O=\log\frac{\exp\left(v_{i}^{c}\cdot v_{j}^{c}\right)}{\sum_{v_{z}\in V}\exp\left(v_{i}^{c}\cdot v_{z}^{c}\right)}. \end{array}$$

Thus, the loss function of generating context embedding can be represented as *ℒ*_*c*_(*e*_*ij*_)=−*𝒪*. With above formulations, CAHNE aims to minimize the overall loss function as

(15) $$\begin{array}{c} ℒ=\sum_{e_{ij}\in E}\left({ℒ}_{s}\left(e_{ij}\right)+{ℒ}_{c}\left(e_{ij}\right)\right). \end{array}$$

At last, the workflow of the context embedding method is summarized in Algorithm 2.

### 4.4. Optimization of CAHNE

#### 4.4.1. Attention for Context Node Sequence

Noticing the context embedding-generating strategy in equation (13), the vector representation of the anchor node *v*_*i*_ is decomposed as the affinity between *v*_*i*_^*t*^ and its context nodes' representations ∑_*j*=1_^*n*^*𝒲*_*i*,*j*_*v*_*i*,*j*_^*t*^. Intuitively, the affinity between context nodes and the anchor nodes should depend on the specific anchor node. For instance, *v*_*i*_ and *v*_*j*_ are anchor nodes in a real-world network, but they have different properties; as a result, they have varied intensity of affinity with their context nodes. Therefore, it is a requisite to incorporate such characters of the anchor nodes in modeling the unique excitation effects *α*.

In line with the attention mechanism [25], a novel and popular model for machine translation, we define the weights between the anchor node and its context nodes with the softmax unit as follows:

(16) $$\begin{array}{c} \alpha_{i}=\frac{\exp\left(v_{i}^{t}+\sum_{j=1}^{n}W_{i,j}v_{i,j}^{t}\right)}{\sum_{m^{\prime }}\exp\left(v_{i}^{t}+\sum_{j=1}^{n\prime }W_{m^{\prime },j}v_{m\prime ,j}^{t}\right)}. \end{array}$$

Therefore, equation (13) can be reformulated as

(17) $$\begin{array}{c} v_{i}^{c}=\tanh\left(v_{i}^{t}+\alpha_{i}\overset{n}{\underset{j=1}{\sum }}W_{i,j}v_{i,j}^{t}\right). \end{array}$$

#### 4.4.2. Negative Sampling

For equation (8) and equation (14), CAHNE aims to maximize the conditional probability between *v*_*i*_ and *v*_*j*_, which is computationally expensive because of the softmax function for all nodes. To address this problem, we adopt the method of negative sampling [26] to approximate the objective function as the following form:

(18) $$\begin{array}{c} \log\;\sigma \left(v_{j}^{T}\cdot v_{i}\right)+\overset{n}{\underset{c=1}{\sum }}E_{z∼P\left(v\right)}\left[\log\;\sigma \left(-v_{j}^{T}\cdot z\right)\right], \end{array}$$

where *σ*(*x*)=1/(1+exp(−*x*)) represents the logistic function and *n* is the number of randomly sampled vertices. We set *P*(*v*) ∝ *d*_*v*_^3/4^, where *d*_*v*_ is the out-degree of *v*. At last, we adopt the Adam algorithm [27] for optimizing equation (18) and set the learning rate as 0.001.

## 5. Experiment

In this section, we empirically evaluate the performance of the proposed framework CAHNE.

### 5.1. Dataset Descriptions

In order to comprehensively evaluate the effectiveness of our model CAHNE, we use seven real-world datasets, including two social networks, two citation networks, one language network, one co-occurrence network, and one communication network, for four applications, i.e., network reconstruction, link prediction, node classification, and visualization. The detailed descriptions are listed as follows:

- Zhihu [28] is a network of social relationships which is an online Q&A platform in China. Users follow each other, asking and answering questions on Zhihu. The text information is concerned topics of each user, which is expressed as full text. We filter out 10000 users from Zhihu who have information on concerned topics. The size of the vocabulary is 9035, and the average length of the text is 89. We evaluate this dataset on the link prediction task.
- HEP-TH [8] is a citation network from arXiv. After filtering out the papers without abstract, 1038 papers are preserved. The text information is expressed as full text. The size of the vocabulary is 2970, and the average length of the text is 54. We evaluate these data on the link prediction task.
- Cora (https://linqs.soe.ucsc.edu/data) is also a citation network containing 2708 machine learning papers with text information classified into one of seven classes. The citation network consists of 5429 links. The text information is expressed as full text. The size of the vocabulary is 16426, and the average length of the text is 88. Cora is used for the link prediction task and node classification task.
- BlogCatalog (http://leitang.net/social_dimension.html) is a large social network of online users listed on the BlogCatalog website. There are 39 different categories of labels for this dataset, and each label represents the metadata provided by a user. Since this dataset does not contain text information, it will be evaluated on the node classification task and network reconstruction for CAHNE (without context embedding).
- Wikipedia [29] is a co-occurrence network which contains 2045 nodes, 17981 edges, and 19 different labels. The tf-idf matrix of the Wikipedia dataset describes the text information for this dataset. There are 4973 columns that correspond to 4973 different words. This dataset will be evaluated on the node classification task.
- 20-NewsGroup (http://qwone.com/∼jason/20Newsgroups/) is a collection of approximately 20,000 newsgroup documents, partitioned (nearly) evenly across 20 different newsgroups. We choose the news documents labeled as comp.graphics, rec.sport.baseball, and talk.politics.gums to evaluate our model on the visualization task. There are 1720 pieces of news contained and expressed as full text. The size of the vocabulary is 30127, and the average length of the text is 206. Besides, 20-NewsGroup is a weighted network.
- Email-Enron (https://snap.stanford.edu/data/email-Enron.html) is a communication network that covers the email communication within a dataset. Nodes are email addresses, and edges denote interactions between emails. Text descriptions of this dataset are full email message text. The size of the vocabulary is 29523, and the average length of the text is 149. We filter 6820 nodes and 23968 edges from the original dataset.

The detailed statistics are summarized in Table 1.

### 5.2. Baselines

We consider the following six NE methods to demonstrate the effectiveness and robustness of CAHNE:

- DeepWalk [4]: it adopts truncated random walk and Skip-Gram model to learn node representations.
- LINE [5]: it preserves the first-order and second-order proximity among nodes in the network.
- Node2vec [6]: it proposes a biased random walk based on DeepWalk to learn node representations.
- GraRep [30]: it integrates global structural information of the graph and uses SVD to train the model.
- Naive Combination: we directly concatenate the text feature embeddings learned by the CNN and node representations learned from LINE for network representation. We choose LINE to learn structure embedding because it can exploit both first-order and second-order proximity in the network, which is more comprehensive than DeepWalk and Node2vec.
- TADW [29]: it integrates text features into network embedding by employing matrix factorization.
- TENE [31]: it learns the representations of nodes under the guidance of both the proximity matrix which captures the network structure and the text cluster membership matrix derived from clustering for text information.
- ASNE [32]: it learns representations of nodes by preserving both the structural proximity and attribute (text) proximity.

### 5.3. Experimental Settings

To be fair, we set the embedding dimension *d*=100 for all methods on HEP-TH, Cora, Email-Enron, and 20-NewsGroup. And for Zhihu, BlogCatalog, and Wikipedia, we set *d*=200. For DeepWalk, we set the window size as 10, the walk length as 80, and the number of walks for each node as 10. For LINE, we set the learning rate as 0.001 and the number of negative samples as 5. For Node2vec, we choose the hyperparameters *p* and *q* to obtain the best performance by grid search. For GraRep, we set the maximum matrix transition step *s* as 5. For TENE, we set the parameter of the contribution of text information *α*=10 and the parameter *β* to guarantee the accuracy of the text cluster membership matrix as 10^7^.

For our model CAHNE, we set the number of negative samples as 5 to speed up the training process. Besides, we set *γ*=0.5 and *k*=2 for all datasets. Hereinafter, we use “CAHNE-a” to validate the effectiveness of our method with the attention mechanism, and “CAHNE(w/o context)” denotes CAHNE without incorporating context embedding.

### 5.4. Network Reconstruction

Reconstructing the network and preserving the original network structure are fundamental objectives for network embedding methods. Definitely, we train an NE method to obtain vector representations of nodes and rank pairwise nodes according to the inner product similarities of them. Since the larger similarities mean higher probabilities of existing edges between pairwise nodes, the top ranking pairwise nodes are used to reconstruct the network efficiently. The precision@k [33] is used as the evaluation metric, which is formulated as

(19) $$\begin{array}{c} \text{precision}@k=\frac{{\sum_{i=1}^{k}\xi_{i}}^{}}{k}, \end{array}$$

where *k* is the number of evaluated pairwise nodes and *ξ* is a binary variable. *ξ*_*i*_=1 denotes the *i*-th reconstructed pair of nodes is correct; otherwise, it is wrong.

**Figure 4:**
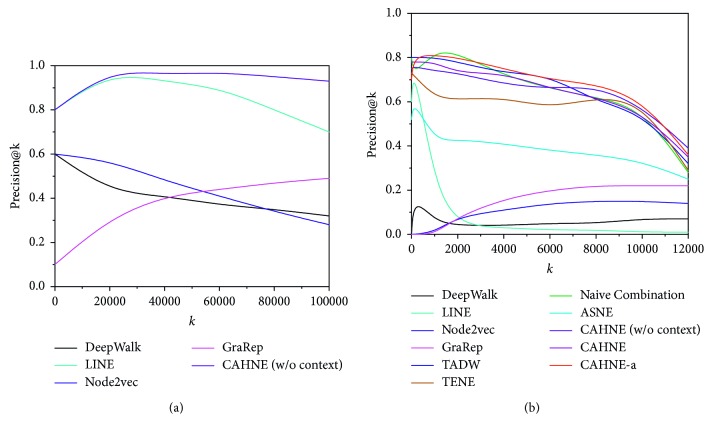
precision@k on (a) BlogCatalog and (b) Email-Enron.

We use a real-world social network BlogCatalog and a communication network Email-Enron as representatives. The result on the precision@k is shown in Figure 4, from which we make the following observations:

- Figure 4 shows that the precision@k of our method CAHNE almost outperforms that of other methods with the increase of *k*, which verifies that CAHNE can perfectly preserve the network structure.
- Because there is no text information in BlogCatalog, Figure 4(a) can clearly reveal that using node importance to weight edges is effective.
- Figure 4(b) shows our method has comparable performance on Email-Enron. We can notice that methods integrating text information are obviously better than other methods, and CAHNE-a can have a relatively high position.

From the above observations, we regard that our method CAHNE and its expansion CAHNE-a achieve a significant advance in efficiency on the task of network reconstruction.

### 5.5. Link Prediction

For link prediction, we use AUC [34] to evaluate the performance, which means the probability that nodes in a random edge are higher than those in a casual nonexistent edge. In this task, as shown in Tables 2–4, we randomly hide certain percentages of edges, respectively, from 85% to 5% on HEP-TH, Cora, and Zhihu and use the left graph to train. We use the logistic regression method to predict the probability of a given pair of nodes has an edge between them.

From these tables, some observations can be listed:

- The results show that the fewer the training edges, the more the nodes are ignored and the lower the performances of all methods. The results on Zhihu are worse than those on other datasets probably because real-world social networks are often accompanied by more complex information from both structures and properties compared to citation networks. However, our proposed model CAHNE-a always achieves the best performances compared to all other baselines on all different datasets. Especially, when the ratio of training edges reaches 95% in Cora and HEP-TH, AUC values of CAHNE-a are higher than 95.
- CAHNE(w/o context) performs better than other structure-only methods (DeepWalk, LINE, Node2vec, and GraRep). It demonstrates that merging node importance when learning network representation is valid and leads to better predicting power for new link formation.
- TADW, TENE, ASNE, and CAHNE perform better than all other structure-only methods. It verifies our assumption that text information cannot be neglected in heterogeneous text networks. However, CAHNE cannot always perform better than TADW, such as shown in 15% in Table 2 and 15% in Table 3. We notice that this phenomenon occurs only when the training ratio is under 35%, which we believe is due to the fact that the CNS cannot contain most context nodes of the anchor node when the training ratio is too low. Also, if the CNS is too incomplete, it will lose a lot of information from the context.  Table 5 shows the average length of CNSs when extracting different ratios of edges as training sets in three datasets. The completeness of CNSs will affect the effectiveness of CAHNE.

Thus, the results in tables can serve as evidence that CAHNE-a has a stable and best performance on all datasets and different training ratios. It demonstrates the flexibility and robustness of CAHNE, and the attention mechanism is significant when learning representations for real-world networks.

### 5.6. Node Classification

**Figure 5:**
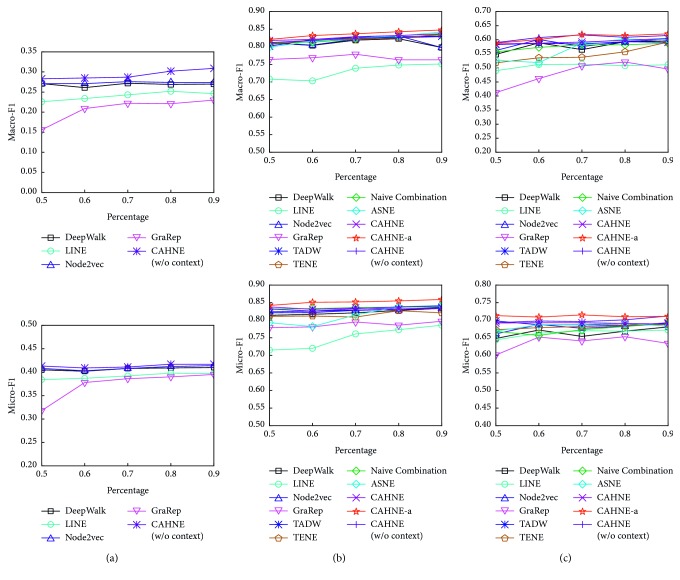
Macro-F1 and Micro-F1 on (a) BlogCatalog, (b) Cora, and (c) Wikipedia.

For this task, we choose BlogCatalog, Cora, and Wikipedia as training datasets in which each node is assigned a label. Given the node embeddings obtained by different NE methods as node features, we train a logistic regression classifier to predict the node labels. We use Macro-F1 and Micro-F1 as measurements to evaluate the performance. We vary the size of the training set from 50% to 90%, and the remaining nodes are the testing set. We repeat each classification experiment ten times and report the average performance in terms of both Macro-F1 and Micro-F1 scores. The results on BlogCatalog, Cora, and Wikipedia are shown and compared in Figure 5. Since BlogCatalog is without text information, we only consider CAHNE(w/o context) on this dataset.

From the results, we obtain the following observations:

- The performances in BlogCatalog are worse than those in other datasets because of the complexity of social networks, and BlogCatalog has the most nodes which could reduce the capability of the classification task, but our proposed model CAHNE(w/o context) still obtains the most satisfactory results.
- For structure-only methods, CAHNE(w/o context) has the best effectiveness on all datasets. It demonstrates that the network representations merging with node importance can be better generalized to the classification task.
- CAHNE(w/o context) performs better than CAHNE and CAHNE-a on Wikipedia as measured by Macro-F1, which indicates this dataset is not sensitive to text information. We believe this is because the text descriptions between different entries vary widely.

### 5.7. Visualization

Another intuitive way to investigate the qualities of network embedding methods is visualization, and in this experiment, we reduce the dimensionality of each representation vector to 2. There are many ways to visualize high-dimensional vectors, e.g., PCA [35], Isomap [9], and t-SNE [36]. In this paper, we adopt t-SNE to achieve dimension reduction because t-SNE can preserve local and global structures of the data. Therefore, we use baselines and our method CAHNE-a to learn representations of the 20-NewsGroup network and input them into t-SNE. From 20-NewsGroup, since all categories of graphs are full connection, to simplify the computational process and improve visualization performance, we filter three categories of news and their documents, comp.graphics, rec.sport.baseball, and talk.politics.gums, as our training set.

**Figure 6:**
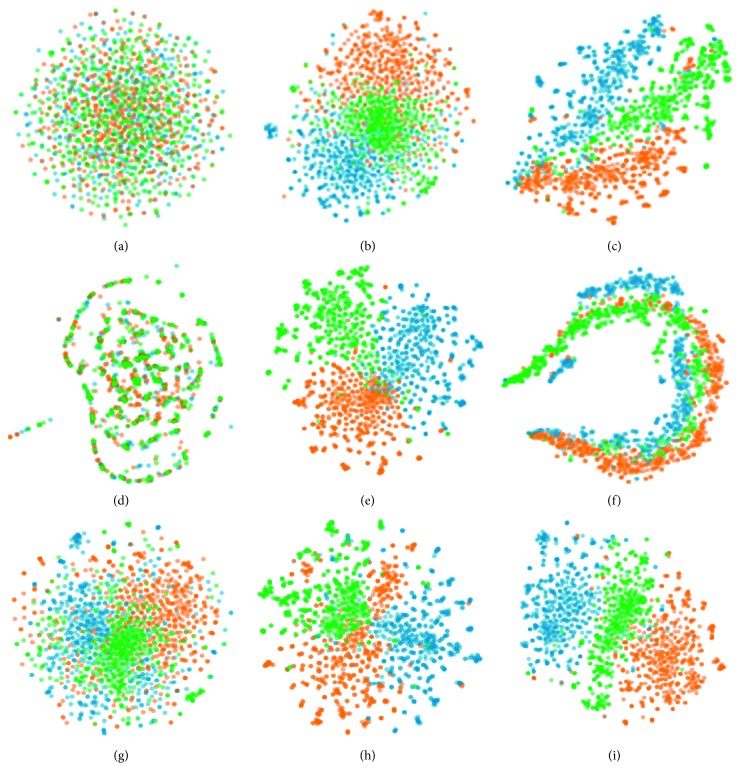
Visualization of the 20-NewsGroup network. Green represents the category of talk.politics.gums, orange represents the category of comp.graphics, and blue represents the category of rec.sport.baseball. (a) DeepWalk. (b) LINE. (c) Node2vec. (d) GraRep. (e) Naive Combination. (f) TADW. (g) TENE. (h) ASNE. (i) CAHNE-a.

The resulting visualizations with baselines and CAHNE-a are illustrated in Figure 6, from which we have the following observations:

- For DeepWalk and GraRep, all points of different categories are chaotic and mixed with each other. Since the network is weighted, DeepWalk cannot handle weighted networks when random walking, which leads to chaos. GraRep integrates weights of edges into representation learning by using E-SGNS, which is powerless to capture the nonlinear relationship between nodes.
- For LINE, ASNE, TENE, and Naive Combination, we can intuitively find the clusters, but the boundary of each category is not clear.
- For Node2vec, we can distinguish three categories more explicitly than for LINE because of a larger space between each cluster. However, the downsides of these clusters are not divisible.
- For TADW, the shapes of clusters are not regular, and the blue points are not getting together.

Obviously, the visualization of our model CAHNE-a has a clear boundary, and the shapes of clusters are more regular than those reported in other baselines.

## 6. Conclusions

In this paper, we propose a novel method to learn node representations for heterogeneous networks, namely, CAHNE. By formulating the context node sequence for each node in a real-world network and redefining the conventional network to integrate text information, CAHNE achieves the learning of node embedding and captures the comprehensive semantic information, maintaining the compatibility between network structures and text information simultaneously. For the unweighted network, we analyze the strength of the relationship between nodes and propose the definition of node importance to quantify it as the weight between nodes. We integrate node importance into the learning process of structure-based embedding to explore the potential structural information in the network. Furthermore, by plugging an attention mechanism in the influence rate of the context nodes, CAHNE obtains the capacity to decide the influence degree from context nodes for different anchor nodes. Extensive experiments prove the competitiveness of CAHNE against baselines and demonstrate the flexibility, stability, and robustness of CAHNE. Future work includes incorporating more types of heterogeneous information like attributes of nodes and edges and optimizing the training process on larger networks.

## Figures and Tables

**Algorithm 1:**
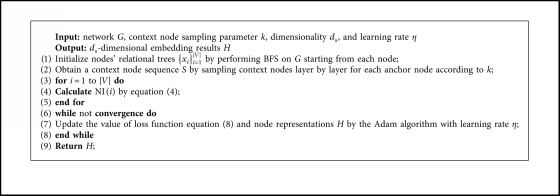
Structure-based embedding with node importance.

**Algorithm 2:**
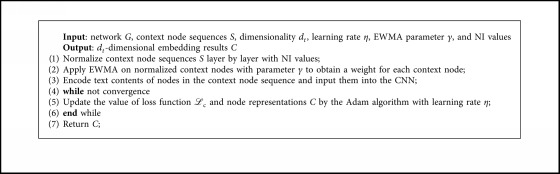
Generating strategy of context embedding.

**Table 1:** Statistics of the dataset.

Dataset	#Nodes	#Edges	#Labels
Zhihu	10000	43894	—
HEP-TH	1038	1990	—
Email-Enron	6820	23968	—
Cora	2708	5429	7
BlogCatalog	10312	333983	39
Wikipedia	2405	17981	19
20-NewsGroup	1720	Fully connected	3

**Table 2:** AUC scores on HEP-TH.

% training edges	15%	25%	35%	45%	55%	65%	75%	85%	95%
DeepWalk	0.658	0.757	0.819	0.864	0.891	0.898	0.897	0.919	0.912
LINE	0.500	0.594	0.717	0.755	0.788	0.806	0.846	0.839	0.923
Node2vec	0.663	0.776	0.845	0.866	0.884	0.899	0.906	0.929	0.915
GraRep	0.628	0.735	0.776	0.841	0.853	0.872	0.885	0.896	0.914
Naive Combination	0.766	0.782	0.788	0.802	0.827	0.856	0.883	0.912	0.928
TADW	0.806	0.818	0.857	0.893	0.902	0.918	0.924	0.936	0.948
TENE	0.778	0.807	0.839	0.862	0.899	0.923	0.928	0.938	0.939
ASNE	0.783	0.802	0.833	0.869	0.893	0.905	0.918	0.926	0.938
CAHNE(w/o context)	0.730	0.796	0.854	0.894	0.893	0.913	0.916	0.921	0.923
CAHNE	0.786	0.818	0.860	0.896	0.902	0.928	0.935	0.937	0.954
CAHNE-a	**0.858**	**0.854**	**0.869**	**0.898**	**0.910**	**0.929**	**0.941**	**0.945**	**0.977**

**Table 3:** AUC scores on Cora.

% training edges	15%	25%	35%	45%	55%	65%	75%	85%	95%
DeepWalk	0.614	0.708	0.777	0.807	0.853	0.858	0.871	0.877	0.898
LINE	0.608	0.743	0.807	0.827	0.853	0.865	0.870	0.885	0.894
Node2vec	0.654	0.722	0.768	0.812	0.838	0.861	0.871	0.878	0.908
GraRep	0.589	0.732	0.786	0.826	0.852	0.874	0.897	0.898	0.914
Naive Combination	0.668	0.772	0.801	0.826	0.852	0.866	0.904	0.921	0.942
TADW	0.803	0.824	0.834	0.862	0.887	0.888	0.903	0.918	0.945
TENE	0.779	0.818	0.822	0.859	0.879	0.881	0.892	0.913	0.916
ASNE	0.718	0.742	0.809	0.832	0.849	0.870	0.902	0.921	0.933
CAHNE(w/o context)	0.654	0.747	0.803	0.843	0.877	0.885	0.901	0.909	0.915
CAHNE	0.793	0.805	0.828	0.863	0.892	0.898	0.908	0.925	0.954
CAHNE-a	**0.805**	**0.830**	**0.837**	**0.872**	**0.892**	**0.907**	**0.915**	**0.926**	**0.963**

**Table 4:** AUC scores on Zhihu.

% training edges	15%	25%	35%	45%	55%	65%	75%	85%	95%
DeepWalk	0.469	0.472	0.497	0.507	0.533	0.537	0.556	0.574	0.587
LINE	0.521	0.569	0.618	0.624	0.655	0.636	0.646	0.676	0.698
Node2vec	0.488	0.482	0.507	0.505	0.552	0.546	0.558	0.582	0.590
GraRep	0.583	0.619	0.642	0.659	0.654	0.662	0.663	0.668	0.663
Naive Combination	0.524	0.553	0.579	0.618	0.653	0.672	0.689	0.705	0.703
TADW	0.558	0.576	0.593	0.625	0.655	0.697	0.696	0.723	0.729
TENE	0.551	0.549	0.607	0.622	0.660	0.666	0.668	0.692	0.711
ASNE	0.586	0.563	0.608	0.633	0.661	0.682	0.699	0.700	0.728
CAHNE(w/o context)	0.595	0.600	0.603	0.604	0.612	0.618	0.639	0.657	0.679
CAHNE	0.623	0.693	0.706	0.709	0.707	0.711	0.713	0.722	0.731
CAHNE-a	**0.631**	**0.707**	**0.721**	**0.724**	**0.723**	**0.727**	**0.736**	**0.748**	**0.759**

**Table 5:** Average length of context node sequences when extracting different ratios of edges.

% training edges	15%	35%	55%	75%	95%
HEP-TH	0.8	2.2	5.7	6.9	7.2
Cora	2.6	4.3	7.9	15.2	17.7
Zhihu	2.3	4.1	6.6	9.7	10.3

## Data Availability

The data used to support the findings of this study are included within the article.
